# Case Report: White–Sutton syndrome and cannabidiol, an update on a reported patient with a successful response to off--label therapy

**DOI:** 10.3389/fped.2025.1515304

**Published:** 2025-02-20

**Authors:** Lorenzo Perilli, Samanta Carbone, Michele Minerva, Margherita Maria Rossi, Maria Rosaria Curcio, Federica Lotti, Salvatore Grosso

**Affiliations:** Clinical Pediatrics, Department of Molecular Medicine and Development, University of Siena, Azienda Ospedaliero-Universitaria Senese, Siena, Italy

**Keywords:** White–Sutton syndrome, cannabidiol, epilepsy, off-label, pediatric neurology

## Abstract

White–Sutton syndrome (WSS), associated with *POGZ* gene mutations, is a rare genetic disorder characterized by a spectrum of phenotypic features, including intellectual disabilities, developmental delays, and epilepsy. A case report described a female patient diagnosed with WSS who experienced seizures resistant to conventional antiseizure medications. Despite various therapeutic attempts, including valproate, topiramate, levetiracetam, clobazam, rufinamide, and vigabatrin, the patient's seizures persisted. After initiating an off-label treatment with cannabidiol (CBD), the patient achieved complete remission from seizures. Following significant clinical improvement, CBD therapy was discontinued by the parents against medical advice, leading to seizure recurrence. Upon reinstatement of CBD, the patient once again experienced successful seizure control. This report emphasizes the need for further investigation into the off-label use of CBD, as an adjunctive therapy in pediatric individuals with drug-resistant epilepsy associated with WSS. Although CBD shows promise in other epileptic syndromes, this case highlights its potential effectiveness in this specific condition. This manuscript aims to contribute to the understanding of WSS and advocate for further research into novel treatments, particularly the role of CBD in managing epilepsy within this complex clinical context.

## 1 Introduction

White–Sutton syndrome (WSS), also known as *POGZ*-Related Intellectual Disability Syndrome, represents a rare genetic disorder characterized by a diverse array of phenotypic features. Since its initial delineation in 2015 by White et al. (1), WSS has garnered increasing attention due to its complex clinical presentation and diverse systemic involvement. This entity has since been associated with mutations in the *POGZ* gene (Pogo Transposable Element Derived with ZNF Domain), which plays a pivotal role in chromatin remodeling and the regulation of gene expression (1). Heterozygous missense, non-sense, and frameshift variants in *POGZ* have been linked to WSS (2, 3). So far, the only non-coding variants associated with this condition affect canonical splice sites or are near these (2–4). Non-sense, frameshift, and splice variants are distributed throughout the gene, with numerous pathogenic variants concentrated in the last exon (2). Although few studies assess these variants at the RNA or protein level (4, 5), some are predicted to cause non-sense-mediated mRNA decay (NMD), while others are expected (or in one case, shown) to evade NMD and produce a truncated protein (2, 4, 5). To date, no clear genotype–phenotype correlations have been established, and its spectrum has not been yet fully delineated (2, 6). This rare condition is characterized by a broad range of intellectual disabilities and global developmental delays, with or without autism spectrum disorder (1–4, 7). Additional phenotypic manifestations, frequently reported, encompass hypotonia, behavioral disorders, ocular issues, variable hearing loss, sleep apnea, microcephaly, and distinctive facial dysmorphisms (2, 3). These dysmorphic features may include broad forehead, midface hypoplasia, triangular mouth, and broad, flat nasal bridge. Additional characteristics may include mild brain abnormalities on imaging, epilepsy, gait abnormalities, brachydactyly, and gastrointestinal issues (6). Furthermore, congenital diaphragmatic hernia (CDH) has been documented in five individuals with heterozygous pathogenic variants in *POGZ* presenting with WSS features (8, 9). In addition, a review of the current literature revealed 18 cases of White–Sutton syndrome with *POGZ* variants and congenital heart disease (10). Epilepsy, as an additional symptom in WSS, has been described in a reported case of a female patient, born and followed up in our center (6). After numerous antiseizure medications (ASMs), an add-on off-label cannabidiol (CBD) therapy resulted in the patient being seizure-free. CBD, an exogenous compound derived from the cannabis plant devoid of psychoactive properties, has emerged as a prospective adjunctive therapy for refractory pediatric epilepsy (11) and for developmental and epileptic encephalopathies (DEE) (12).

Current evidence indicates that patients with a wide variety of epilepsy disorders and underlying causes may experience a positive response to treatment with a highly purified, plant-derived CBD oil solution (13), constituting this as a feasible off-label therapeutic alternative in many other rare pediatric epilepsies (Table 1).

**Table 1:** Narrative review of the use of CBD in epilepsy.

On label	Off label
Dravet syndrome (14)	Monogenic epilepsies including SYNGAP1 (13), CDKL5 (13), SCN8A (13), SHANK1 (15)
Lennox–Gastaut syndrome (14)	Structurally determined epilepsies including lyssencephaly (13), focal cortical dysplasia (13), tumor-related epilepsy (13), cerebral dysgenesis (13), polymicrogyria (16)
Tuberous sclerosis complex (17)	Chromosomic imbalances including Dup15q (13), ring chromosome 20 (16), Ring chromosome 17 (16), 12q trisomy (18)
	Syndromes including Aicardi (13), Doose (13), Sturge–Weber (13), Rett (13) infantile/epileptic spasms (13) Lafora (16), Unverricht–Lundborg (16), fragile X (19)

An extensive body of preclinical and clinical investigations has illuminated CBD's antiseizure properties mediated through modulation of neurotransmitter release, ion channel activity, and inflammatory pathways (20, 21). Although the precise mechanistic underpinnings of CBD's efficacy in epilepsy remain incompletely understood, accumulating evidence substantiates its utility as a promising therapeutic option for mitigating drug-resistant seizures across a spectrum of epileptic syndromes and DEE.

Pharmacoresistance in POGZ variants has not been quantified, although it is frequently reported (6). Moreover, seizure control is often considered one of the major factors influencing the quality of life in patients with epileptic syndromes, thus achieving it remains among the foremost goals. Therefore, it is essential to implement efficacy studies of individual therapies in this condition (22, 23). Our aim with this manuscript is highlighting the need for further investigations regarding the off-label use of CBD in this rare condition.

## 2 Case report

Ferretti et al. (6) reported a 5-year-old female patient affected by WSS, carrying a heterozygous c. 2711T > G variant in the *POGZ* gene. The child was born at 37 weeks of gestational age via induced labor following spontaneous membrane rupture. A normal gestational and familial history was reported. The toddler suffered neonatal distress requiring respiratory support. Shortly after birth, she showed poor reactivity, spontaneous mobility, visual impairment, and feeding difficulties, requiring Neonatal Intensive Care Unit. Furthermore, she exhibited dysmorphic features such as microcephaly, diffuse hair in the frontal region, temporal narrowing, broad nasal bridge, pointed chin, bifid uvula, mild clinodactyly of the fifth finger (right > left), and bilateral prominent calcanei. Feeding difficulties persisted associated to moderate gastric distension. At 4 months of age, the toddler experienced paroxysmal episodes mainly while awake, isolated or in clusters, characterized by hyperextension of the upper limbs and psychomotor arrest with eyes deviation, lasting from 10 to 30 s. In the suspect of non-epileptic episode, considering the absence of electroencephalogram (EEG) correlates and normal brain MRI, clobazam was introduced without benefit. Subsequently, hands/feet motor stereotypies and extrusion reflex appeared. At 2 years old, a focal seizure with hypertonus of the upper limbs with inter ictal epileptiform EEG abnormalities occurred. Thereafter spasms and myoclonic seizures emerged with a frequency of several per month. During the years various ASMs were administered: valproate, topiramate, levetiracetam, rufinamide, clobazam, and vigabatrin were introduced without success. These medications were often combined and, in certain instances, discontinued from the therapeutic regimen due to insufficient efficacy or the emergence of adverse effects.

Within the course of the various follow-ups in our center, at 6 years old, along with the other mentioned ASMs, an off-label add-on CBD treatment at the dosage of 10 mg/kg/day was initiated resulting in a complete clinical remission from seizures. In the presence of complete resolution, no significant changes were observed in the EEG. Cannabidiol is approved as a labeled treatment, showing good efficacy in terms of seizure control and cognitive outcome, in various epileptic encephalopathies such as Dravet syndrome, Lennox–Gastaut syndrome, and tuberous sclerosis complex. Considering the well reported (12, 15) efficacy in many other monogenic epilepsy syndromes (Table 1), after counseling with the patient's family, we decided on a therapeutical off-label attempt. Prior to the initiation, the family was adequately informed about the objectives and the risks of the off-label therapy and provided their written consent. After the titration of CBD, the patient went from experiencing multiple episodes per month to complete resolution. At 9 years old, the parents discontinued CBD therapy due to significant clinical improvement, against medical advice. Shortly thereafter, focal motor seizures with impaired awareness characterized by upper limb hyperextension recurred. Consequently, CBD therapy was successfully reinstated, confirming the important clinical intervention of this ASM, in this child.

**Figure 1:**
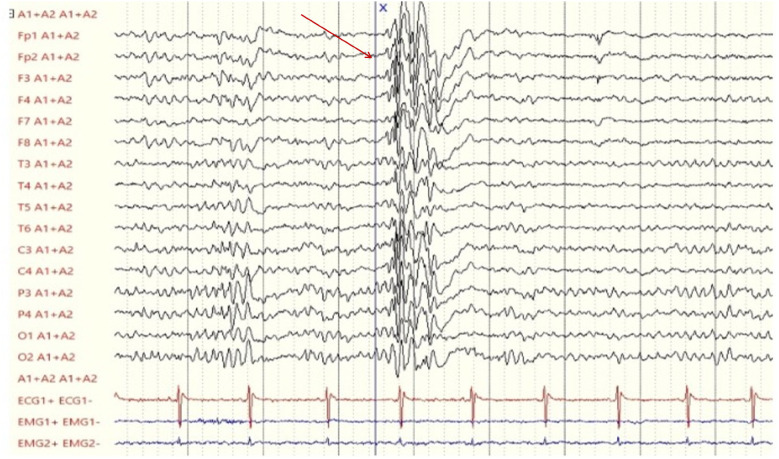
Last electroencephalogram (performed on the patient at 12 years and 4 months of age) showing a diffuse paroxysm of epileptiform abnormalities (red arrow), characterized by polyspikes and spike-wave complexes with low recurrence, present during wakefulness and sleep, without clinical correlation.

The latest follow-up at 12 years and 4 months of age showed poor neurodevelopmental outcome (she establishes eye contact, sits independently, but there is no verbal production or autonomous walking) with persistence of the seizure-free state. The medication regimen with CBD has not been modified in terms of dosage, maintaining the initial dose per kilogram. At the last follow-up, the EEG (Figure 1) reported interictal diffuse paroxysm without clinical evidence in a well-structured regional differentiation trace.

## 3 Discussion

The clinical presentation of WSS encompasses a broad spectrum of features including autism spectrum disorder, developmental delay, intellectual disability, and epilepsy (6). Furthermore, commonly observed characteristics entail feeding and gastrointestinal disorders, sleep disturbances, genitourinary abnormalities, and hearing and visual impairment. Given the limited efficacy of conventional ASMs in WSS-associated epilepsy, there is growing interest in exploring alternative therapeutic treatments.

CBD has emerged as a promising on-label adjunctive therapy for epilepsy in Dravet, Lennox–Gastaut, and tuberous sclerosis complex syndromes, demonstrating its antiseizure properties and positive outcomes on cognitive functions (24), as well as an off-label option in other refractory epilepsies and DEE (11, 12, 20). Among the reviewed scientific literature, to our knowledge, this patient is the first reaching a consistent seizure-free period with an add-on off-label CBD therapy. Realizing CBD's therapeutic promise requires focused efforts to establish its long-term safety and efficacy. The evidence of its importance as a potential target for the development of new antiepileptic drugs comes from animal rather than human studies, and the growing available data will shed light on the mechanisms of action of CBD in modulating endocannabinoid tone, thereby explaining its reported efficacy in epilepsy.

CBD, a non-psychoactive component of cannabis, exhibits a complex mechanism of action contributing to its antiepileptic effects. Its pharmacological profile is likely polypharmacological, involving modulation of several targets including the equilibrative nucleoside transporter, orphan G-protein-coupled receptor (GPR55), and various transient receptor potential channels (TRPM8, TRPA1, TRPV1, TRPV2). CBD enhances 5-HT1a receptor activity and glycine receptors (α3 and α1), while influencing intracellular calcium dynamics.

Notably, CBD's lipophilic properties allow it to access intracellular sites, particularly mitochondria, which may play a role in its neuroprotective effects. CBD also modulates [Ca2+] levels in various cell types, including hippocampal neurons, and its actions are negatively modulated by the endocannabinoid system. At higher concentrations, CBD activates nuclear peroxisome proliferator-activated receptor-*γ* and inhibits the degradation of the endocannabinoid anandamide. In addition, its polyphenolic nature provides antioxidant, antiapoptotic, and anti-inflammatory effects by modulating cytokine release and glial cell function.

Specifically, as proposed by Rosenberg et al., CBD seems to restore the balance between excitatory and inhibitory signals in the hippocampus by blocking the effects of the lysophosphatidylinositol (LPI) at the GPR55 receptor. Considering that seizures rapidly enhance the GPR55-LPI pathway and at the light of the above-mentioned CBD effect on this route, this could provide a potential explanation for CBD's anticonvulsant effects in dampening hyperexcitability (25).

CBD is metabolized mainly by the enzymes CYP2C19 and CYP2C9, with CYP3A4 involved in other metabolic processes. The key active metabolite, 7-hydroxy-CBD, is primarily formed by CYP2C19, with a contributing role from CYP2C9. Interestingly, the production of 7-OH-CBD does not seem to be affected by different CYP2C19 gene variants, and the impact of polymorphisms in CYP3A4 and CYP2C9 has not been studied. The metabolism of ASMs primarily occurs via hepatic pathways, with interindividual variability, playing a key role in determining both pharmacokinetic and pharmacodynamic responses (26). A possible explanation to the good response to CBD could be related to the patient's specific metabolism, leading to better efficacy in this individual.

Given these multifaceted actions, CBD shows promise as an anticonvulsant treatment for resistant epilepsy in children (11).

Although a wide range of other ASMs could have been considered viable options, taking into account the risk–benefit ratio and our experience with CBD in similar drug refractory epileptic patients, we deemed it the most appropriate alternative.

While this case remains anecdotal and it represents a limitation to the present report, we believe it is noteworthy due to the remarkable therapeutic response observed, peculiarly when interrupted and promptly reintroduced, warranting further exploration of CBD's potential in similar contexts. Even if the clinical response was striking, considered that to date no precise interreferences of CBD on the etiopathogenesis of WSS have been described, the short-term follow-up and lack of data on long-term outcomes are significant constraints, underlining the need for larger cohort studies to validate these finding and elucidate the underlying pathophysiology of epilepsy in WSS, exploring novel therapeutic interventions.

Other limitations to the present case could be represented by placebo effect and parental reporting. Nonetheless, considered the fact that CBD therapy was introduced after a long-standing history of ineffective pharmacological treatments, the subsequent follow-up in other centers, where the patient was reported to be seizure-free, and the reliability of the family, we consider unlikely that the clinical improvement could be solely attributed to placebo or parental reporting.

Discontinuing CBD against medical advice also involves significant ethical and practical considerations in pediatric off-label therapies. Medical teams should approach adherence challenges with sensitivity, ensuring clear communication with parents about the risks and benefits of continuing or stopping treatment. Collaborative decision-making, addressing concerns, exploring alternatives, and considering the child's wellbeing and family values are essential. Support systems, including counseling and multidisciplinary care, can also help improve adherence and build trust between families and healthcare providers. Our aim with this article is to provide other feasible therapeutic interventions in drug-resistant WSS individuals.

## Data Availability

The original contributions presented in the study are included in the article/Supplementary Material, further inquiries can be directed to the corresponding author.
